# A Novel Antibody-Based Biomarker for Chronic Algal Toxin Exposure and Sub-Acute Neurotoxicity

**DOI:** 10.1371/journal.pone.0036213

**Published:** 2012-05-02

**Authors:** Kathi A. Lefebvre, Elizabeth R. Frame, Frances Gulland, John D. Hansen, Preston S. Kendrick, Richard P. Beyer, Theo K. Bammler, Frederico M. Farin, Emma M. Hiolski, Donald R. Smith, David J. Marcinek

**Affiliations:** 1 Exposure Assessment and Biomedical Models, Northwest Fisheries Science Center, National Marine Fisheries Service, NOAA, Seattle, Washington, United States of America; 2 The Marine Mammal Center, Sausalito, California, United States of America; 3 U. S. Geological Survey-Western Fisheries Research Center, Seattle, Washington, United States of America; 4 Department of Environmental and Occupational Health Sciences, University of Washington, Seattle, Washington, United States of America; 5 Department of Microbiology and Environmental Toxicology, University of California Santa Cruz, Santa Cruz, California, United States of America; 6 Departments of Radiology and Bioengineering, University of Washington, Seattle, Washington, United States of America; University of New South Wales, Australia

## Abstract

The neurotoxic amino acid, domoic acid (DA), is naturally produced by marine phytoplankton and presents a significant threat to the health of marine mammals, seabirds and humans via transfer of the toxin through the foodweb. In humans, acute exposure causes a neurotoxic illness known as amnesic shellfish poisoning characterized by seizures, memory loss, coma and death. Regular monitoring for high DA levels in edible shellfish tissues has been effective in protecting human consumers from acute DA exposure. However, chronic low-level DA exposure remains a concern, particularly in coastal and tribal communities that subsistence harvest shellfish known to contain low levels of the toxin. Domoic acid exposure via consumption of planktivorous fish also has a profound health impact on California sea lions (*Zalophus californianus*) affecting hundreds of animals yearly. Due to increasing algal toxin exposure threats globally, there is a critical need for reliable diagnostic tests for assessing chronic DA exposure in humans and wildlife. Here we report the discovery of a novel DA-specific antibody response that is a signature of chronic low-level exposure identified initially in a zebrafish exposure model and confirmed in naturally exposed wild sea lions. Additionally, we found that chronic exposure in zebrafish caused increased neurologic sensitivity to DA, revealing that repetitive exposure to DA well below the threshold for acute behavioral toxicity has underlying neurotoxic consequences. The discovery that chronic exposure to low levels of a small, water-soluble single amino acid triggers a detectable antibody response is surprising and has profound implications for the development of diagnostic tests for exposure to other pervasive environmental toxins.

## Introduction

Domoic acid (DA) is a small water-soluble marine algal toxin that is naturally produced by diatoms of the genus *Pseudo-nitzschia* during harmful algal blooms (HABs) [1]. In humans, acute exposure causes a neurotoxic illness known as amnesic shellfish poisoning (ASP) that is characterized by gastrointestinal distress, seizures, confusion, memory loss, coma and death in the most severe cases [2]. The first documented cases of ASP occurred in Eastern Canada in 1987 when over 100 people became ill and 3 died as a result of consuming DA contaminated mussels [3]. Since that event, consistent monitoring of shellfish beds for the presence of DA in edible shellfish tissues (≥20 µg DA/g shellfish = regulatory limit) has been effective in protecting human consumers from acute DA exposure [4]. However, there are growing concerns about the impacts of low-level repetitive exposure, particularly in coastal and tribal communities that rely heavily on the harvests of local shellfish with known low levels of the toxin [5]. In addition to human health concerns, DA exposure via consumption of planktivorous fish has a significant impact on California sea lions along the US West Coast resulting in the stranding and/or deaths of hundreds of animals yearly [6], [7]. These naturally exposed sea lions represent a valuable “sentinel” species for human health risks as related to DA exposure. Recently, two syndromes of DA toxicosis have been identified in sea lions, an acute syndrome and a chronic syndrome [8]. The chronic syndrome has been observed in stranded sea lions even in the absence of DA producing algal blooms in the environment, suggesting that sub-lethal DA exposure leads to lasting neurologic effects in mammalian species [8].

In light of the recognized increase in frequency and geographic range of HABs globally [9], there is a critical need for reliable diagnostic tests for assessing chronic HAB toxin exposure in coastal human populations and wildlife. To date, most suspected DA toxicosis cases are investigated by behavioral observations, post mortem histologic examination of brain tissue, and examining bodily fluids, stomach contents and/or feces for the presence of the toxin [6], [7], [10], [11]. None of these methods are effective for accurately assessing chronic low-level exposure and the associated sub-acute neurotoxic effects, which are the main human health related concerns [12]. For example, behavioral symptoms typically represent high-level exposures and examination of brain tissues for damage is possible only after death or with expensive MRI techniques in live animals [13]. Additionally, systemic toxin levels are not reliable indicators of exposure because DA is rapidly depurated, with 99% of circulating DA levels excreted within 4 hours in experimentally injected primates [14]. Therefore toxin levels or lack thereof are not indicative of exposure levels or history. The lack of a diagnostic biomarker for chronic DA exposure presents a significant barrier to properly characterizing exposure and health risk, because without the ability to assess exposure, there is no way to accurately determine health and disease impacts related to chronic exposure.

In the present study, zebrafish were used as a vertebrate model species to identify potential health risks and associated biomarkers of chronic low level DA exposure applicable to human and marine mammal populations. Chronic low-level exposure to DA induced an antibody response and increased toxin sensitivity. Historically, specific antibody responses have been generated in other vertebrate species against small haptens that are similar in size to DA when coupled to serum proteins for immunogenicity [15]. Importantly, the putative antibody response in zebrafish was further validated in field exposed California sea lions. Thus we demonstrate that this antibody response is an ecologically relevant biomarker of chronic exposure and increased neurologic sensitivity that may be useful for assessing DA related sublethal pathology in both marine mammal and human populations.

## Methods

### Chronic Exposures in Zebrafish

Wild-type zebrafish (*Danio rerio*, AB strain) were obtained from Oregon State University, Sinnhuber Aquatic Research Laboratory (Corvallis, OR) at approximately 5 months of age and maintained at the Northwest Fisheries Science Center (NWFSC) in a ZebTec stand-alone recirculating and continuously monitored zebrafish rack system with UV sterilizer (Techniplast, Exton, PA). Fish were maintained at 26°C, kept on a 12∶12 hr light∶dark cycle, and fed daily with commercially available fish feed. At approximately 7 months of age, zebrafish were repetitively exposed to asymptomatic doses of DA via intracoelomic (IC) injection for 36 weeks, followed by a seven-week recovery period. Blood serum (n = 9 control, n = 9 exposed) and whole brains (n = 9 control, n = 9 exposed) were collected from zebrafish at 12 time points (6, 24 and 48 hours, and 1, 2, 6, 12, 18, 24 and 36 weeks of exposure and 3 and 7 weeks of recovery) throughout the experiment for DA quantification in serum and whole brain transcriptome analyses via microarray. Toxin and vehicle (PBS) injections were given once a week (dose = 0.31±0.03 µg DA/g fish) for the first 6 weeks, then once every two weeks (dose = 0.18±0.02 µg DA/g fish) for the duration of the exposure period of 36 weeks. Doses of less than half of the previously reported EC_50_ (0.86 µg DA/g fish) for zebrafish [16] were chosen to ensure asymptomatic exposures. All toxin dose concentrations were verified via HPLC as described in Lefebvre et al. (2009) [16]. Domoic acid injections were performed as described in Lefebvre et al. (2009) [16].

### Zebrafish Serum Collection and Analyses

For serum collection at all 12 time points, zebrafish (n = 9 control and n = 9 exposed) were individually netted and placed in an ice bath until quiescent. The tail was then cut posterior to the anal fin and blood was collected from the caudal vein using a heparinized 44.7 µl microcapillary tube (Drummond Scientific Company, Broomall, PA). Tubes were sealed at one end with clay and spun in a hematocrit centrifuge for one minute to separate plasma from red blood cells. Tubes were scored at the interface between plasma and red blood cells and the plasma was collected in a microfuge tube and stored at -20°C until analysis.

Domoic acid was quantified in three serum replicates (each consisting of 3 pooled zebrafish serum samples) using a commercially available competitive ELISA kit (Biosense®). DA values were quantified following the kit guidelines at the following time points; 6, 24, and 48 hours, and 1, 2, 6, 12, 18, 24 and 36 weeks of exposure, and at 3 and 7 weeks after the last injection (recovery time points). It was later discovered that although the commercial kit was designed and advertized for DA quantification exclusively, the competitive ELISA format detects the presence of both DA and DA-specific antibodies indiscriminately in serum (see results section).

### Brain Dissections and RNA Extraction

Brains were dissected as described in Lefebvre et al. (2009) [16]. Global transcriptome expression was quantified via microarray in three RNA replicates (each consisting of RNA from 3 pooled brains) for exposed and control treatments. Test group size was chosen based on a previous study in which the appropriate number of zebrafish brains required to obtain sufficient quantities of high quality RNA for microarray analysis was determined [16]. Total RNA was isolated from zebrafish brains (n = 9 for each treatment and time point) using the miRNeasy Mini Kit (Qiagen Inc., Valencia, CA) according to the vendor's defined method, and stored at −70°C. The quantity (ng/uL) of RNA was determined measuring the OD_260_ with a NanoDrop ND-1000 Spectrophotometer (Thermo Fisher Scientific, Waltham, MA) and the RNA purity was assessed measuring OD_260/280_ and OD_260/230_ ratios. RNA integrity (quality) was characterized using the Agilent RNA 6000 Nano Kit with an Agilent 2100 Bioanalyzer (Agilent Technologies, Santa Clara, CA). Only total RNA samples with appropriate size distribution, quantity, and A260∶A280 as well as A260∶A230 ratios of 1.8–2.1 were used for microarray-based analysis. Individual brain RNA samples were split and half used for microarray analyses and half used for RT-PCR confirmation.

### Global Transcriptome Profiling in Brain

The RNA samples were labeled and prepared for hybridization onto a Zebrafish (V3) Gene Expression Microarray (Agilent Technologies, Inc. Santa Clara, CA) using the manufacturer's established protocols. Hybridization and washing of these arrays was accomplished using HS 400 Pro hybridization and wash stations (Tecan Systems, Inc., San Jose, CA) and scanned using an Agilent DNA Microarray Scanner (Agilent Technologies, Inc. Santa Clara, CA) using previously established methods. In total, 72 microarrays were used, including 36 for the control group and 36 for the chronic exposure group. Separate pools of RNA from three individuals were hybridized to each array resulting in three biological replicates (a total of nine individuals) per treatment group and time point.

### Microarray Analyses

Raw microarray data was generated with the Agilent Feature Extraction image analysis software (Agilent). Raw microarray data were further processed and analyzed with tools from Bioconductor [17]. Data were normalized using the BioconductorAgi4×44PreProcess package [18]. Additionally, the normexp option for background adjustment and quantile normalization was used for the between array normalization step. Using the normalized data, we identified genes with significant evidence for differential expression using the limma package. The limma methodology calculates a P-value for each gene using a modified t-test in conjunction with an empirical Bayes method to moderate the standard errors of the estimated log-fold changes. This method of detecting differentially expressed genes draws strength across genes for more robust and accurate detection of differentially expressed genes. Such an adjustment has repeatedly been shown to avoid an excess of false positives when identifying differentially expressed genes [19]. Using the P-values from limma, we used the Bioconductor package p.adjust [20] to estimate the false discovery rate associated with our list of differentially expressed genes. This methodology allows us to address the multiple testing problem without resorting to an excessively conservative approach that controls the familywise error, such as a Bonferroni correction. In addition, we used the Bioconductor kmeans [21] function from the stats package to perform K-means clustering of the genes. In order to identify Gene Ontology categories that were enriched with the differentially expressed genes, we used the functional annotation clustering tool available through NIAD DAVID (http://david.abcc.ncifcrf.gov/) [22].

### Quantitative RT-PCR Analyses

**Figure 1:**
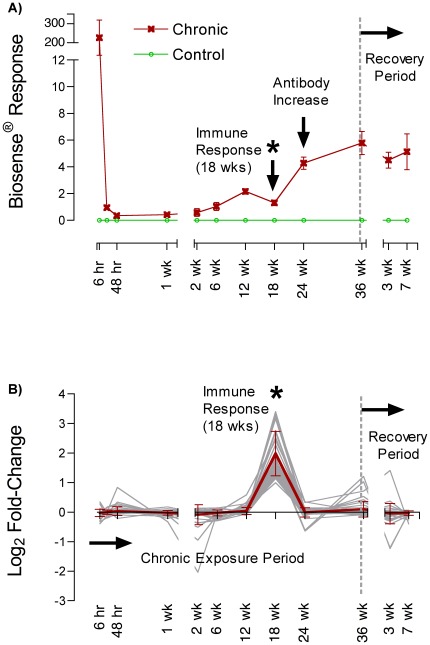
A) Response of Biosense® (Bergen, Norway) competitive enzyme-linked immunosorbent assays (ELISAs) in serum of exposed and time-matched control zebrafish (*Danio rerio*) at all sampling time points. Detection by competitive ELISA indicates the presence of DA and/or DA-specific antibodies in serum. **B**) One K-means cluster for a set of significantly differentially expressed genes (1.5 fold; p<0.05) quantified in whole brains from exposed and control zebrafish. The y-axis represents the log_2_ fold-change for genes in this cluster at all sampling time points (x-axis). The K-means algorithm forces all differentially expressed genes into clusters or groups with other genes that behave most similarly over all time points. Once clusters have been formed, further analysis of the known functions of the genes within each cluster can be used to identify potentially impacted biological processes. Functional annotation clustering using DAVID (http://david.abcc.ncifcrf.gov/) of the genes shown here identified Immune Function as the major functional group (enrichment score: 1.98). Up regulation of immune related genes at 18-weeks (demarked by *) indicates a significant immune response, after which antibody levels appear to rise in the blood (Figure 1A).

Four immune-relevant genes displaying significant upregulation by microarray analyses at 18 weeks in the K-means cluster shown in Figure 1B were further validated by RT-PCR at four time points (6, 12, 18, and 24 weeks). Total RNA was isolated from individual zebrafish brains (n = 9 control and n = 9 chronic at four time points as described above). RT-PCR was performed on individual samples. Briefly, reverse transcription was performed according to the manufacturer's established protocol using total RNA and the SuperScript® III First-Strand Synthesis System (Invitrogen, Carlsbad, CA.). For gene expression measurements, 2 mL of cDNA were included in a PCR reaction (12 mL final volume) that consisted of the ABI inventoried TaqMan® Gene Expression Assays mix (Applied Biosystems Inc., Foster City, CA), or customized forward and reverse primers, probes and TaqMan Gene Expression Master Mix (Applied Biosystems Inc., Foster City, CA). The PCR primers and the dual-labeled probes for the genes were designed using ABI Primer Express v.1.5 software (Applied Biosystems Inc., Foster City, CA). Amplification and detection of PCR amplicons were performed with the ABI PRISM 7900 system (Applied Biosystems Inc., Foster City, CA) with the following PCR reaction profile: 1 cycle of 95°C for 10 min, 40 cycles of 95°C for 30 sec, and 60°C for 1 min. Beta-actin 1 amplification plots derived from serial dilutions of an established reference sample were used to create a linear regression formula in order to calculate expression levels, and Beta-actin 1 gene expression levels were utilized as an internal control to normalize the data.

### Dose Response Assays to Assess Neurologic Sensitivity

In order to quantify the impact of chronic exposure on neurologic sensitivity, a second chronic exposure experiment was performed in zebrafish for 20 weeks under the same chronic low-level exposure conditions as described for the first experiment. At 20 weeks, chronically exposed zebrafish and control zebrafish were subjected to a dose response bioassay. Dose response assays were performed and ED_50_ calculations made as described in Lefebvre et al. (2009) [16] with the exception that five DA doses and a PBS control dose were used in the present study. Spiral swimming was used as the behavioral metric to determine acute neurotoxicity. All doses were validated using HPLC [16].

### Indirect ELISAs for Domoic acid-Specific Antibody Detection in Sea Lion Serum

Sea lion serum samples from 17 naturally exposed animals admitted to The Marine Mammal Center were analyzed for the presence of a DA-specific IgG antibody via an indirect ELISA (Table 1). Additionally, five control serum samples were obtained from healthy sea lions born and raised in captivity at Sealife Park Hawaii for method validation. Detection specificity of DA-specific sea lion IgG was accomplished using a DA conjugated 96-well plate. Serum was applied to sample wells and DA-specific antibodies allowed to bind to the DA conjugated to the plate. After thorough washing, HRP labeled anti-canine IgG was allowed to incubate, TMB substrate was added, and absorbance was detected with a Bio Tek Epoch Plate reader. The indirect format of this ELISA detects DA specific antibodies exclusively and is not subject to “false positive” interference by the presence of DA.

**Table 1:** Confirmation of the presence of a domoic acid (DA)-specific antibody in naturally exposed California sea lion (*Zalophus californianus*) serum samples as detected via an indirect enzyme-linked immunosorbent assay (ELISA).

CSL-ID #	Absorbance Ratio	Seizures (yes/no)	Admit Date	Serum Collection Day^a^
W-9759	9.88	Yes	7/10/10	1
W-9864	7.59	Yes	10/11/10	0
W-9876	2.85	Yes	10/16/10	1
W-10047	1.72	Yes	8/11/11	1
W-6920	1.51	No	6/30/06	1
W-7923	1.27	No	11/10/08	49
W-10046	1.23	Yes	8/11/11	1
W-6990	1.23	No	10/16/06	7
W-10035	1.16	Yes	8/5/11	2
W-10045	1.12	Yes	8/10/11	1
W-10044	1.11	Yes	8/10/11	1
W-9114	nd	Yes	10/7/09	18
W-8739	nd	Yes	7/21/09	4
W-10026	nd	Yes	8/5/11	3
W-8032	nd	No	1/16/09	32
W-6980	nd	No	8/15/06	3
W-7899	nd	No	10/15/08	35
C- 1	nd	No	na	na
C- 2	nd	No	na	na
C- 3	nd	No	na	na
C- 4	nd	No	na	na
C- 5	nd	No	na	na

CSL = California sea lion; W = Wild; C = control healthy animals born and raised in captivity at Sealife Park Hawaii with presumably no history of domoic acid exposure; Absorbance Ratio = sample serum absorbance÷(mean control serum absorbance+3SD); Values >1 indicate presence of DA-specific antibody; nd = not detectable; na = not applicable;

a Number of days after admission to the Marine Mammal Center before serum was collected.

## Results

### Zebrafish Serum Analyses via Biosense® Competitive ELISAs

Serum DA values as quantified via Biosense® ELISA in control and exposed zebrafish are depicted in Figure 1A. The first time points (6 hrs, 24 hrs, 48 hrs and 1 week) represent a depuration curve for DA in blood serum after the first injection for the study. Serum samples for time points 2 through 36 weeks were all taken one week after the most recent DA injection. The final two data points to the right of the dashed line represent recovery time points (3 and 7 weeks after the last DA injection). In spite of the fact that DA doses and dosing frequency decreased after 6 weeks, toxin values in serum as quantified via Biosense® ELISA increased. Additionally, these values remained high throughout the recovery period up to 7 weeks after the last DA injection (Figure 1A). Further examination of the “competitive” ELISA format revealed that the commercial assay detects both DA and DA-specific antibodies. The fact that DA is highly water soluble and rapidly excreted, led us to interpret that the increase in DA signal after 18 weeks and into recovery was likely due to the presence of a DA-specific antibody and not an indication of increased serum DA levels (Figure 1). For example, a DA specific antibody (IgM) present in zebrafish serum would bind to DA conjugated to the ELISA plate and compete with the Biosense® labeled detection antibody, resulting in a decrease in absorbance just as DA in a sample competes with the labeled antibody in solution, also causing decreased absorbance. These data provided the first indication that chronic low-level DA exposure elicits a detectable antibody response in the zebrafish model even in the absence of overt neurobehavioral excitotoxicity.

### Whole Brain Transcriptome Analyses in Zebrafish

Data from whole brain transcriptome analyses provided further evidence for the induction of a significant immune response at the later chronic exposure time points. K-means clustering of all significantly differentially expressed genes (1.5 fold; p<0.05) in whole brains taken from zebrafish at all time points identified a cluster dominated by immune function genes (DAVID; http://david.abcc.ncifcrf.gov/; enrichment score: 1.98) that were significantly upregulated at 18 weeks, just before the apparent increase in DA specific antibody titer in serum (Figure 1B). Gene Ontology (GO) terms for Biological Process represented in the annotation clusters included “antigen processing and presentation”, “MHC protein complex” and “immune response”.

### RT-PCR Confirmation of Significantly Differentiated Genes

Four immune-relevant genes (CC chemokine (CCL19-like), MHC class IIA and B, and an MHC class I gene) displaying significant upregulation by microarray analyses and present in the K-means cluster shown in Figure 1B were further validated by RT-PCR using B-actin as a control at four time points (6, 12, 18 and 24 weeks). For the 16 contrasts (four genes at four time points), 14 agreed in fold change direction while two were too small in magnitude for reliable comparisons.

### Dose Response Analyses and Neurologic Sensitivity

**Figure 2:**
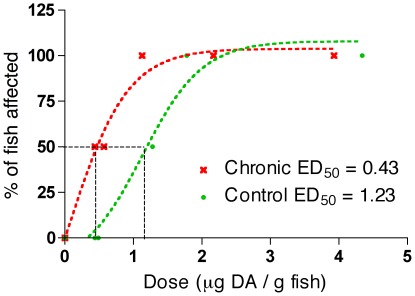
Dose-response relationship between intracoelomic (IC) injection doses of domoic acid (DA) and the percentage of zebrafish affected (n = 4 at each dose) for chronically exposed (red line) and control (green line) zebrafish. Circular and/or spiral-swimming behaviors were used to quantify excitotoxicity in fish. The 50% Effective Dose (ED_50_) = the dose at which 50% of the fish tested were affected (µg DA/g). The dose-response experiment was carried out at 20 weeks of exposure (one week after the last injection).

Dose response curves for chronically exposed zebrafish and naively exposed zebrafish are shown in Figure 2. The 50% effective dose (ED_50_) is calculated from the dose response relationship as the dose that effects 50% of the test population and is used as a metric for comparing toxin sensitivities. Resulting ED_50_s of 0.43 and 1.23 µg DA/g for chronic and naive populations, respectively, indicated that chronically exposed zebrafish were three times more sensitive to DA than naively exposed zebrafish.

### Antibody Detection in Sea Lion Serum via Indirect ELISAs

Eleven of 17 wild sea lions admitted to The Marine Mammal Center had detectable levels of DA-specific antibodies in serum, thereby verifying the presence of the proposed biomarker in a naturally exposed mammalian species. Five control sea lion serum samples from captive animals yielded a mean absorbance value of 0.051±0.002 (SD). An additional blank (n = 6) using sample buffer only (no serum) yielded a mean absorbance value of 0.049±0.002 (SD). Together these data confirmed that non-specific binding of sea lion IgG in control serum did not occur in this assay format. Absorbance ratios greater than one indicate the presence of DA-specific antibodies (Table 1). Absorbance ratios were calculated by dividing the wild serum sample absorbance (X) by the mean control serum absorbance plus three times SD (X/0.057).

## Discussion

Together our data revealed that chronic low-level exposure to DA induces a detectable antibody response and has underlying neurotoxic consequences. The sub-acute repetitive dosing regime in the zebrafish provided a vertebrate model that identified a diagnostic biomarker of chronic exposure that was further verified in a naturally exposed mammalian human health sentinel species, the California sea lion.

At the transcriptional level, whole-genome microarray profiling provided evidence of a significant immune response in the zebrafish model that was temporally linked to the proposed antibody response (Figure 1). Gene Ontology (GO) terms for Biological Process represented in the annotation clusters included “antigen processing and presentation”, “MHC protein complex” and “immune response”, all of which are suggestive of an activated immune system. Four immune-relevant genes from this cluster displaying significant upregulation by microarray analyses were further validated by RT-PCR; CC chemokine (CCL19-like), MHC class IIA and B, and an MHC class I gene. The altered expression of these genes is likely derived from chronically activated microglia, the resident macrophages of the brain, resulting in enhanced proinflammatory responses, chemotaxis, and antigen presentation in the brain [23], [24]. Chemokines are small, secreted proteins that function in leukocyte trafficking and activation but are also associated with inflammatory and pathological processes [25] as might be expected from chronic DA exposure (e.g. neuronal degeneration). Additionally, upregulation of MHC class I and II genes implies enhanced antigen presentation of non-self to cytotoxic and helper T-cells, respectively. Taken together, the induction of these and other immune-relevant genes implies that chronic DA exposure results in immune activation. It is unlikely that DA alone would result in lymphocyte activation, but when coupled to carrier proteins in serum or on cells, it could modify self-proteins and thus act as a potent hapten for stimulating both B and T cell mediated responses including specific antibody production. Hapten modified self-proteins have been shown to elicit antibody production and T cell effector function [26], [27], [28], [29]. Specifically, a potential mechanism for DA mediated humoral responses observed in this study could include DA modification of self through reactive carbonyl groups on DA as shown for other small haptens [30].

In addition to a DA specific immune response, chronic low-level DA exposure had quantifiable neurologic consequences on whole animal health. In dose response experiments, chronically exposed zebrafish were three times more sensitive to DA than naively exposed zebrafish (ED_50_s of 0.43 and 1.23 µg DA/g for chronically-exposed and naive populations, respectively), revealing that repetitive low-level exposure increases neurologic sensitivity to the toxin in subsequent exposures (Figure 2). This increased toxin sensitivity has profound health implications for human populations that regularly consume shellfish containing low levels of DA as well as for repetitively exposed sea lions. The results suggest that long-term low-level exposure does not result in tolerance or resistance but actually increases the health risks associated with subsequent exposures. This finding and its linkage to a detectable DA specific antibody response provide a valuable example of a diagnostic test specific for chronic low-level exposure that is linked to a subclinical neurotoxic health condition.

Further testing in wild naturally-exposed California sea lions confirmed that the proposed DA specific antibody response identified in zebrafish is a potential biomarker for chronic DA exposure in natural “at risk” populations. Utilizing an anti-canine IgG antibody that cross-reacts with sea lion IgG [31] and a DA-conjugated plate, we developed an indirect ELISA that exclusively detects DA-specific antibodies in sea lion serum without interference by DA. The presence of DA specific antibodies was confirmed in 65% of the sea lion serum samples analyzed (n = 11 of 17; Table 1). Veterinarians frequently use the presence of seizures to diagnose symptomatic DA toxicosis, once other common causes of seizures such as trauma, infections and metabolic imbalances have been ruled out [7]. Based on data from the zebrafish model, repetitive exposure over time is required for induction of a DA specific antibody response (Figure 1A). This indicates that the presence of antibodies selectively identifies chronically exposed animals and that antibodies may not be present in naive animals or those without sufficient repetitive exposure. In our sea lion data set, three animals (W-6920, 7923, and 6990) had detectable DA-specific antibody titers, but had not exhibited clinical signs of DA-induced excitotoxicity (e.g. seizures), suggesting a chronic exposure history, but no recent acute exposure. Conversely, three other animals (W-9114, 8739, and 10026) exhibited DA-related seizures, but did not have measurable quantities of DA-specific antibodies (Table 1), suggesting that these animals had been acutely exposed based on clinical signs of toxicity but potentially not chronically exposed based on lack of detection of the antibody biomarker. The distinction of “chronic” exposure for sea lion health assessments is critical for prescribing proper treatments as chronically exposed animals have different health changes and expected recovery outcomes than acutely exposed sea lions [8]. In the case of “at risk” human populations, it is chronic low-level exposure that presents the most significant health risk and the absence of a biomarker for exposure has been a barrier to accurately assessing the health and disease impacts related to chronic exposure in these populations [12].

### Conclusions

Collectively, our data provide strong evidence for the production of a specific antibody response induced by repetitive low-level exposure to a pervasive algal toxin. The resulting antibody response fills a critical need for a quantifiable biomarker for chronic exposure that would be useful for wildlife as well as human health assessments. The next steps are to validate this finding in human populations, particularly those with regular exposure to low levels of DA. Several coastal and tribal communities in the Pacific Northwest region of the U.S. rely heavily on razor clams as a food source, a species known to accumulate DA and retain the toxin for long periods [32]. The biomarker discovered here identifies chronic exposure exclusively and not acute one time exposure. This type of biomarker is critically important for human health assessments of pervasive environmental toxins, especially in light of our finding that chronic exposure increases toxin sensitivity, thereby making organisms more susceptible to DA toxicity in subsequent exposures. This comprehensive chronic exposure study, leading from the identification of an antibody-based biomarker in a laboratory fish model to field verification of the biomarker in naturally exposed mammals provides a new paradigm for chronic exposure assessment that can be applied to other pervasive environmental toxins.
